# Retrospective Evaluation of Bone Metastases in Patients With Thyroid Malignancy: A Single-Center Experience

**DOI:** 10.7759/cureus.52079

**Published:** 2024-01-11

**Authors:** Müge Yaşar, Ensar Aydemir, Coşkun Ateş, Filiz Mercan Sarıdaş, Erhan Hocaoğlu, Buğra Taygun Gülle, Özen Öz Gül, Soner Cander, Erdinç Ertürk, Canan Ersoy

**Affiliations:** 1 Department of Endocrinology and Metabolism, Bursa Uludağ University Faculty of Medicine, Bursa, TUR; 2 Department of Endocrinology and Metabolism, Edirne Sultan Murat I State Hospital, Edirne, TUR; 3 Departments of Public Health and Epidemiology, Dokuz Eylul University, İzmir, TUR

**Keywords:** medullary thyroid cancer, differentiated thyroid cancer, thyroid pathology, bone metastases, thyroid cancer

## Abstract

Background

Thyroid cancer is one of the five most common cancers causing bone metastasis. If there is an increase in serum thyroglobulin-antithyroglobulin levels in differentiated thyroid cancer or calcitonin levels in medullary thyroid cancer, patients should be evaluated for recurrence and distant metastasis. The skeleton is the second most common site of distant metastasis in thyroid cancer after the lung. Bone metastases cause pain, fractures, and spinal cord compression, severely reducing the quality of life. They are associated with poor prognosis. Bone metastases severely reduce the quality of life. This study aimed to retrospectively evaluate the diagnosis and follow-up of patients with thyroid cancer with bone metastases diagnosed at our center.

Methodology

A total of 1,390 patients diagnosed with thyroid malignancy at our center between 2010 and 2023 were reviewed retrospectively. The study included 27 patients with differentiated and medullary thyroid cancer who had bone metastases.

Results

Of 27 patients, 19 (70.4%) had differentiated and eight (29.6%) had medullary thyroid cancer. Papillary thyroid cancer constituted 22.2% (*n* = 6) and follicular thyroid cancer constituted 14.8% (*n* = 4) of the cases. Papillary carcinoma follicular variant, oncocytic, and poorly differentiated thyroid cancer were diagnosed with similar frequency, each accounting for 11.1% (*n* = 3). It was found that vertebrae were most commonly involved, followed by the pelvis, sternum, costae, femur and patella, shoulder and humerus, cranium, and scapula. The five-year survival rate was 72%, and the 10-year survival rate was 53%.

Conclusions

The number of patients with papillary cancer was the highest, but the rate of bone metastases was the lowest in this group. The highest rate of bone metastases was found in patients with poorly differentiated, oncocytic, medullary, follicular, and papillary cancer, respectively. The results obtained in this study reveal the necessity and importance of bone metastasis evaluation in patients with thyroid cancer.

## Introduction

Bone metastases occur in approximately 2%-5% of all thyroid cancer patients [1,2]. Thyroid cancer is one of the five most common cancers causing bone metastasis. Among differentiated thyroid cancers, follicular thyroid cancer has a higher risk of bone metastasis than papillary cancer (7%-28% vs. 1.4%-7%, respectively) [3,4]. In medullary thyroid cancer, bone metastasis has been reported at rates ranging from 20% to 40% [5,6]. The skeleton is the second most common site of distant metastasis after the lung [2]. Bone metastases cause pain, fractures, and spinal cord compression, severely reducing the quality of life [1,2,7]. They are associated with poor prognosis. Methods such as direct radiography, computed tomography (CT), magnetic resonance imaging (MRI), whole-body bone scintigraphy, and positron emission tomography (PET) are used to detect bone involvement in metastatic thyroid cancer or visualize the detected lesions in detail. Surgery is a treatment option but not suitable for all cases [8,9]. Radiation therapy (RT), tyrosine kinase inhibitors (TKIs), and ablative treatments play an important role in treatment and palliative care. Anti-resorptive agents, including bisphosphonates and denosumab, are used in treatment [10]. This study aimed to retrospectively evaluate the diagnosis, treatment, and follow-up results of patients with bone metastases diagnosed with differentiated and medullary thyroid cancer at our center.

## Materials and methods

After obtaining approval from the ethics committee (decision numbered 2011-KAEK-26), the files of 1,390 patients diagnosed with thyroid malignancy at our center between 2010 and 2023 were retrospectively reviewed. A total of 27 patients with differentiated and medullary thyroid cancer with bone metastases were included in the study. Patients with anaplastic thyroid cancer and concomitant differentiated and medullary thyroid cancer were excluded. SPSS Statistics for Windows, Version 29 (IBM Corp., Armonk, NY) was used for statistical data analysis. For descriptive statistics, mean, standard deviation, median, minimum, and maximum values were calculated for continuous variables and number and percentage values were calculated for discrete variables. The Mann-Whitney U test and the chi-square test were used to compare nonparametric data between the two groups. The Spearman correlation test was used to evaluate the correlation of nonparametric data. The results were assessed at a 95% confidence interval, and statistical significance was defined as *P* < 0.05.

## Results

Of the 27 patients included in the study, 15 (55.5%) were female and 12 (44.4%) were male, with a mean age of 60.2 ± 12 years (min-max = 33-78). Nineteen (70.4%) cases had differentiated thyroid cancer, and eight (29.6%) cases had medullary thyroid cancer. The rate of bone metastases in 1,390 patients was 1.9%, compared to 0.7% in 1,287 cases of papillary thyroid cancer, 11.1% in 36 cases of follicular cancer, 18.6% in 43 cases of medullary thyroid cancer, 33.3% in 9 cases of oncocytic cancer, and 42.8% in 7 cases of poorly differentiated thyroid cancer. When the pathological diagnoses of thyroid cancers with bone metastasis were analyzed, medullary thyroid cancer (*n* = 8, 29.6%) was the most common cancer metastasizing to the bone. Papillary thyroid cancer constituted 22.2% (*n *= 6), and follicular thyroid cancer constituted 14.8% (*n* = 4) of the cases. Papillary carcinoma follicular variant, oncocytic, and poorly differentiated thyroid carcinoma were diagnosed with similar frequency, each accounting for 11.1% (*n* = 3).

The median time to bone metastasis was 2 (min-max =1-15) years for differentiated thyroid cancer and 3.5 (min-max = 1-14) years for medullary thyroid cancer. No significant difference was observed between the two groups (*P* = 0.477).

The mean primary thyroid tumor size at diagnosis was 48 mm, with a median value of 50 mm. There was no significant correlation between tumor size at diagnosis and time to bone metastasis (*P* = 0.439). Pathological evaluation of the cases revealed lymphovascular invasion in 92.6% of cases.

Initial detection of bone metastases was achieved through MRI (29.6%), bone scintigraphy (25.9%), PET-CT (22.2%), CT (18.5%), and whole-body iodine scintigraphy (WBIS) (3.7%).

**Figure 1:**
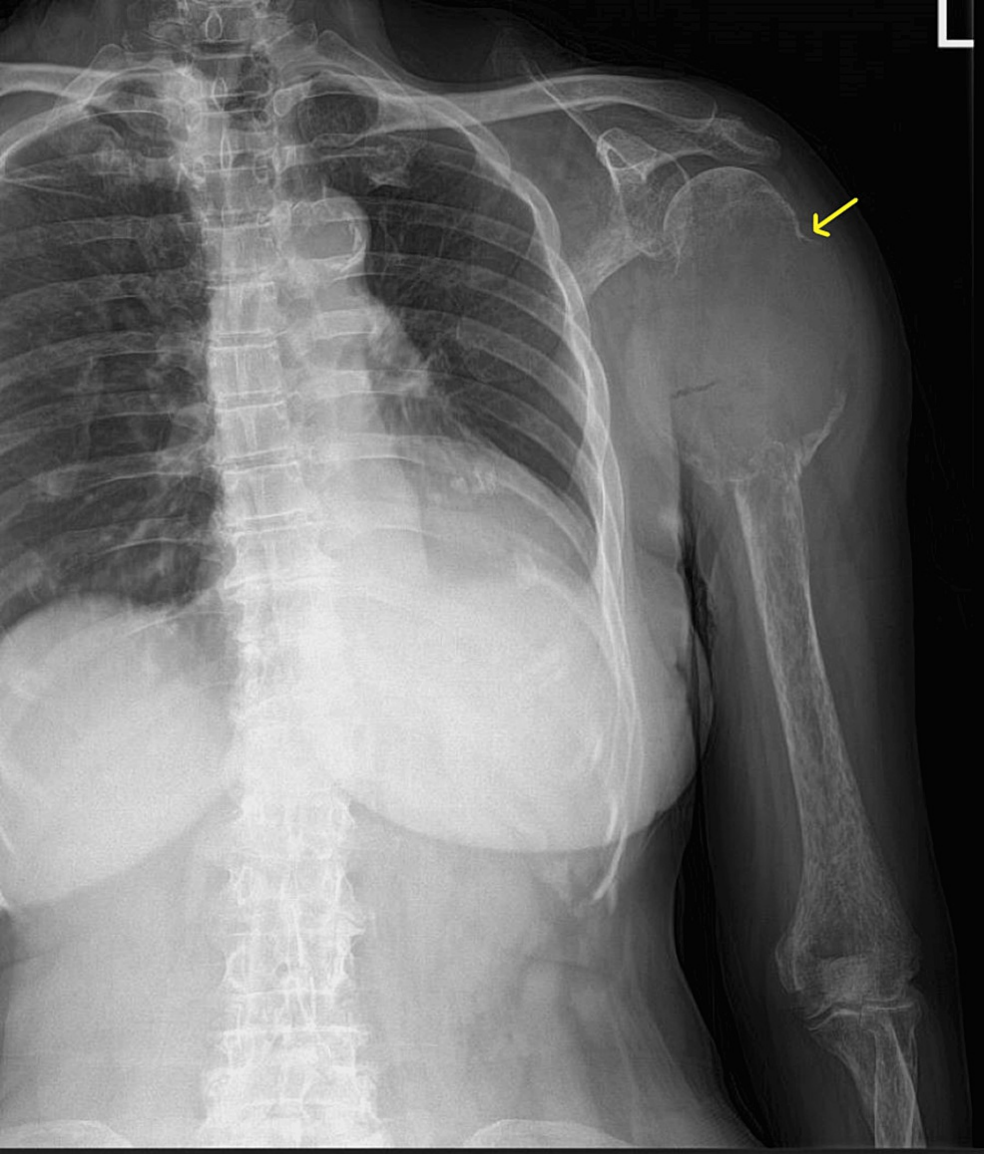
A 73-year-old female patient with oncocytic cancer presented with left shoulder-humerus metastasis, as observed on a direct radiograph.

**Figure 2:**
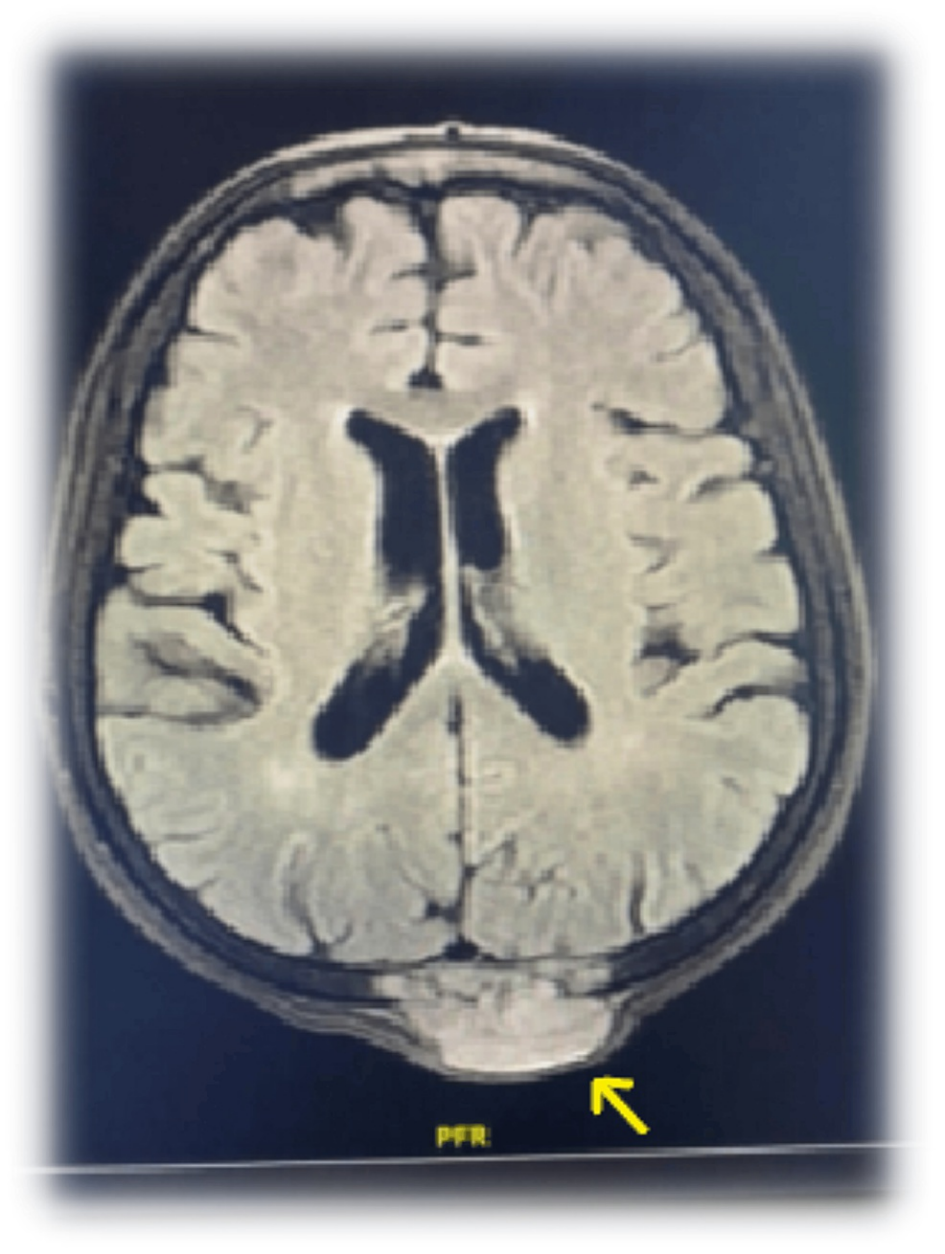
A 73-year-old female patient with oncocytic cancer exhibiting cranium metastasis, as evidenced by magnetic resonance imaging (MRI).

In three patients, bone metastases were detected using imaging methods, and the thyroid cancer was diagnosed through biopsies from the pelvis (*n* = 2) and sternum (*n* = 1). All other patients had bone metastasis complications at follow-up after detecting thyroid malignancy. During follow-up, large painful masses were found in one patient’s cranium, humerus, and knee region, which were also detected through physical examination (Figures 1-2). A humeral biopsy performed for differential diagnosis of osteosarcoma for the second primary cancer was compatible with metastasis of oncocytic cancer, a primary thyroid cancer.

When the localization of bone metastases was evaluated, it was found that vertebrae (77.8%), pelvis (55.6%), sternum (33.3%), costae (25.9%), femur and knee (14.8%), shoulder and humerus (14.8%), cranium (3.1%), and scapula (3.1%) were involved. Metastases in more than one skeletal site were seen in 19 (70.4%) patients. Multiple bone metastases were detected in two sites in seven (25.9%) patients, three sites in eight (29.6%) patients, four sites in three (11.1%) patients, and five sites in one (3.7%) patient.

Symptoms and signs associated with skeletal metastases, such as severe pain, fracture, spinal cord compression, and malignant hypercalcemia, were present in 66.7% of patients. Two patients had vertebral fractures due to bone metastases. One patient developed neurogenic bladder and urinary incontinence due to vertebral involvement.

There was no statistically significant correlation between skeletal metastasis-related complications and age, gender, and number of skeletal sites with metastasis (*P* = 0.706; *P* = 0.338; *P* = 0.068, respectively).

Skeletal metastasis-related complications were seen in three of eight patients with medullary thyroid cancer and 14 of 19 patients with differentiated and poorly differentiated thyroid cancer. There was no significant difference between the two groups in the incidence of skeletal metastasis-related complications (*P* = 0.102) and the number of skeletal sites with metastases (*P* = 0.585).

**Figure 3:**
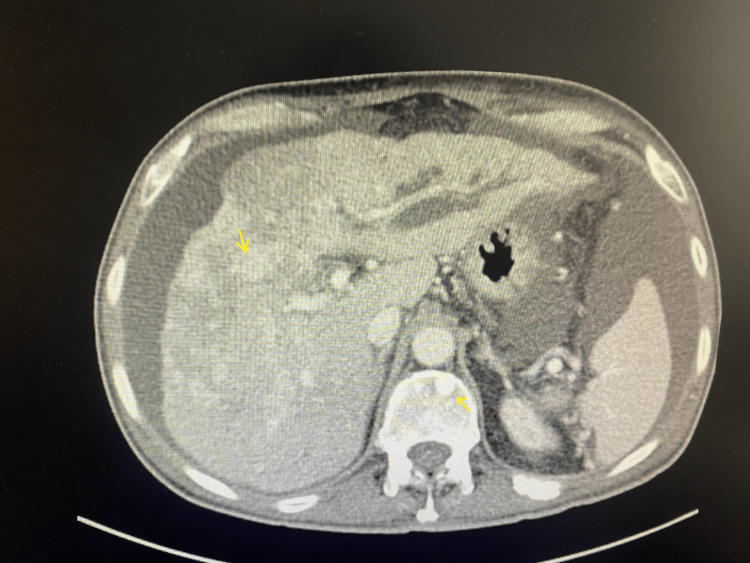
Liver and vertebral metastases in a 70-year-old male patient with medullary cancer (computed tomography).

When the patients were analyzed in terms of nonskeletal organ metastases upon detecting bone metastasis, 25 (92.6%) patients had neck lymph node metastasis, 21 (77.8%) patients had lung metastasis, and five (18.5%) patients had liver metastasis. One of the metastases was liver metastasis due to differentiated thyroid cancer, while all other metastases were associated with medullary thyroid cancer (Figure 3).

Patients with differentiated thyroid cancer were evaluated for radioactive iodine therapy (RAI) after bone metastasis, and eight (42.1%) eligible patients received RAI. Differentiated and medullary thyroid cancer bone metastases were treated with radiotherapy (RT; 66.7%) and surgery (25.9%). Two patients underwent vertebroplasty, while surgical excision was performed in the sternum in two patients, in the costa in one patient, in the pelvis in one patient, and in the shoulder in one patient.

Nineteen (70%) patients received tyrosine kinase inhibitor treatment. Sorafenib was used in 12 patients with differentiated thyroid cancer, cabozantinib was used in three patients with differentiated thyroid cancer, and vandetanib was used in five patients with medullary thyroid cancer. In one patient, sorafenib, vandetanib, and cabozantinib treatments were administered consecutively in different periods, while in another patient, vandetanib and cabozantinib treatments were administered. In one patient, bosutinib treatment was considered appropriate because of the concomitant diagnosis of chronic myeloid leukemia. Six patients received cytotoxic chemotherapy: four with cyclophosphamide vincristine dacarbazine (medullary), one with carboplatin-paclitaxel (medullary), and one with doxorubicin (poorly differentiated). All patients with bone metastases were treated with zoledronic acid once every three months.

The five-year survival rate was 72%, and 10-year survival rate was 53%. Of the 27 patients with bone metastases, seven (25.9%) died in a median time of five years after diagnosis. The earliest death occurred within one year. There was no statistically significant association between mortality and gender, age, and skeletal-related complications (*P* = 1.000, *P* = 0.150, and *P* = 0.678, respectively). Of the patients with a mortal prognosis, three patients were diagnosed with poorly differentiated, one patient with medullary, one patient with papillary, one patient with follicular variant of papillary carcinoma, and one patient with a follicular thyroid cancer. All patients with poorly differentiated cancer and bone metastasis had a mortal outcome.

## Discussion

In this study, we retrospectively evaluated the diagnosis, treatment, and follow-up of 27 patients with bone metastases who were followed up in our center with differentiated and medullary thyroid cancer diagnoses.

Bone metastases occur in approximately 2%-5% of all thyroid cancer patients. In this study, this rate was found to be 1.9%, close to the lower limit of the literature data [1]. The mean time to bone metastasis reported in the literature was one to four years. This was consistent with the data obtained in this study [11]. The average age of bone metastasis in the literature was 60 years. The female/male ratio was close to 1. The mean tumor size was 40-50 mm. The median age, gender distribution, tumor size at the time of diagnosis, and mean time to bone metastasis in our patients with thyroid malignancy and bone metastases were found to be consistent with the literature [10,12].

Studies have shown that tumor size at diagnosis has prognostic significance [13]. However, the relationship between tumor size and time to bone metastasis has not been evaluated. In this study, no significant correlation was found between these two parameters.

In a study of 44 patients with primary thyroid pathology and bone metastasis, 45.4% had follicular cancer, 36.3% had papillary cancer, 6.8% had anaplastic cancer, 6.8% had medullary cancer, and 4.5% had oncocytic cancer [14]. In another study, 33.6% of the patients had follicular cancer, 24.5% had papillary cancer, 22.4% had a follicular variant of papillary cancer, 9.1% had oncocytic cell cancer, and 10.5% had differentiated cancer aggressive histology or poorly differentiated cancer [15]. In some studies, the metastasis rate was three times higher in follicular thyroid cancer compared to papillary thyroid cancer [14]. In another study, bone metastasis was five times more common in follicular cancer than in oncocytic cancer [16]. However, the rates obtained in this study differed from the literature. This can be explained by the small sample size. Among thyroid cancers, papillary thyroid cancer remains the most common histological subtype, followed by follicular, medullary, poorly differentiated, and anaplastic thyroid cancer [17]. In this study, papillary cancer was the most common malignancy, but the rate of bone metastasis was the lowest in this group. The highest rates were observed in the patient groups diagnosed with poorly differentiated, oncocytic, medullary, and follicular cancers, respectively.

MRI has been reported to have the highest sensitivity for the initial detection of bone metastases, followed by CT, PET-CT, bone scintigraphy, and WBIS. Sensitivity is lower on direct radiography [1]. In this study, the initial detection of bone metastases was mostly achieved by MRI. This was followed by other imaging methods. In this study, scans were mostly performed with bone scintigraphy, the second most common method for the initial detection of bone metastasis.

In another study, it was reported that 52% of bone metastases were detected before the diagnosis of thyroid cancer [14]. In this study, bone metastases were detected before the diagnosis of thyroid cancer in three (11%) patients.

Thyroid cancers frequently metastasize to the axial skeleton, especially the vertebrae. In other studies, vertebral metastases were seen more frequently. Other sites of metastasis are the pelvis, thorax, extremities, shoulder, and craniomaxillofacial bones, respectively [7,18,19]. The distribution of skeletal metastasis sites is similar in our patient group. In other studies, multiple skeletal metastases have been reported in approximately 50%-60% of cases [9,19]. In this study, the rate of metastasis in more than one skeletal region was 70.4%.

Bone metastases cause pain, fractures, and spinal cord compression, severely reducing the quality of life [20]. They are associated with poor prognosis. Our cases were also complicated with complaints of pain. There were also more serious complications, such as urinary incontinence, ileus, and vertebral fractures. The frequency of skeletal metastasis-related complications is consistent with the literature (66.7%). Studies have reported rates of up to 70%. Although it has been reported that there is no significant correlation between skeletal-related complications and the number of skeletal foci with metastasis, the results obtained in this study showed that complications were more frequent as the number of metastasis foci increased [11]. Consistent with the literature, there was no difference between differentiated and medullary thyroid cancer in terms of skeletal-related complications [4]. No association was found between skeletal-related complications and mortality in this study.

In patients with thyroid malignancy, distant metastases often affect multiple organs, including the lung, liver, and bones. As was the case in our patients, bone metastases are highly associated with lung metastases [5].

In patients with bone metastases, external RT can be applied for palliative purposes and pain control. Due to its ability to reduce tumor size, external RT is an effective treatment for severe pain and spinal cord compression [3].

Surgery can be considered in bone metastases if the lesion is isolated and suitable for surgical excision [9]. Surgery can be used alone or in combination with RAI and kinase inhibitors to treat skeletal lesions in selected patients [21,22]. RAI resistance is observed in distant metastasis of differentiated thyroid carcinoma. Skeletal metastases in differentiated thyroid cancer are largely unsuitable for RAI treatment [23,24]. This rate was similar in this study.

Systemic therapy is recommended in RAI-resistant disease, and tyrosine kinase inhibitors (TKI) are used [23,25]. In our patients, tyrosine kinase inhibitor (TKI) therapy was also incorporated into the treatment. For patients with extensive bone metastases, zoledronic acid infusion therapy, a bisphosphonate, is recommended once every three months, particularly if TKI therapy is to be administered or if the patient is already undergoing treatment [23]. Denosumab is an alternative to bisphosphonate therapy for treating thyroid cancer patients with bone metastases [10]. In this study, all patients received zoledronic acid treatment.

Studies have reported a five-year survival rate of 70%-80% and a 10-year survival rate of approximately 40%-50% in thyroid cancer patients with bone metastases [14]. Survival rates were similar in this study. All patients with poorly differentiated cancer and bone metastasis had a mortal outcome. No patient with oncocytic cancer died.

## Conclusions

In conclusion, bone metastases in thyroid cancer can be detected in approximately half of patients with distant metastasis. The skeleton is the second most common site of distant metastasis after the lung. Screening for lymph node and lung metastases is common in thyroid cancers while screening for bone metastases is less common. The results obtained in this study reveal the necessity and importance of bone metastasis evaluation in patients with thyroid cancer.
